# Reconstruction of Ancestral Gene Orders Using Probabilistic and Gene Encoding Approaches

**DOI:** 10.1371/journal.pone.0108796

**Published:** 2014-10-10

**Authors:** Ning Yang, Fei Hu, Lingxi Zhou, Jijun Tang

**Affiliations:** 1 Tianjin Key Laboratory of Cognitive Computing and Application, Tianjin University, Tianjin, China; 2 Department of Computer Science and Engineering, University of South Carolina, Columbia, South Carolina, United States of America; 3 School of Automation, Northwestern Polytechnical University, Xi'an, shaanxi, China; Wilfrid Laurier University, Canada

## Abstract

Current tools used in the reconstruction of ancestral gene orders often fall into event-based and adjacency-based methods according to the principles they follow. Event-based methods such as GRAPPA are very accurate but with extremely high complexity, while more recent methods based on gene adjacencies such as InferCARsPro is relatively faster, but often produces an excessive number of chromosomes. This issue is mitigated by newer methods such as GapAdj, however it sacrifices a considerable portion of accuracy. We recently developed an adjacency-based method in the probabilistic framework called PMAG to infer ancestral gene orders. PMAG relies on calculating the conditional probabilities of gene adjacencies that are found in the leaf genomes using the Bayes' theorem. It uses a novel transition model which accounts for adjacency changes along the tree branches as well as a re-rooting procedure to prevent any information loss. In this paper, we improved PMAG with a new method to assemble gene adjacencies into valid gene orders, using an exact solver for traveling salesman problem (TSP) to maximize the overall conditional probabilities. We conducted a series of simulation experiments using a wide range of configurations. The first set of experiments was to verify the effectiveness of our strategy of using the better transition model and re-rooting the tree under the targeted ancestral genome. PMAG was then thoroughly compared in terms of three measurements with its four major competitors including InferCARsPro, GapAdj, GASTS and SCJ in order to assess their performances. According to the results, PMAG demonstrates superior performance in terms of adjacency, distance and assembly accuracies, and yet achieves comparable running time, even all TSP instances were solved exactly. PMAG is available for free at http://phylo.cse.sc.edu.

## Introduction

### Overview

Evolutionary biologists have had a long tradition in reconstructing genomes of extinct ancestral species. Mutations in a genomic sequence are made up not only at the level of base-pair changes but also by rearrangement operations on chromosomal structures such as inversions, transpositions, fissions and fusions [2]. Over the past few years, ancestral gene-order inference has brought profound predictions of protein functional shift and positive selection [3].

Methods for ancestral genome reconstruction either assume a given phylogeny that represents the evolutionary history among given species or search the most appropriate tree along with a set of ancestral genomes to fit the observed data. Depending on how the gene-order data is interpreted and handled, methods for solving the latter can be partitioned into two groups: event-based methods and adjacency-based methods. Event-based methods typically search for the set of ancestral gene orders that minimizes the sum of rearrangement distances over all edges of the given phylogeny. However, methods seeking exact solutions following such paradigm (such as GRAPPA [4], MGR [5], [6]) have already encountered huge difficulties in handling modern genomes due to their NP-hard complexity. In consequence, methods such as GASTS [7] were developed to provide heuristic solutions. In addition to event-based methods, a number of adjacency-based methods have been proposed such as SCJ [8], InferCARsPro [9] and GapAdj [10]. Instead of explicitly considering a predefined set of rearrangement events, these methods take gene adjacencies into account and treat them as binary characters with present and absence states. In this way, by viewing the gene order as a set of gene adjacencies, the goal is to determine which adjacencies are contained in the genome.

We recently developed an adjacency-based method in the probabilistic framework called PMAG [1] to reconstruct ancestral genomic orders given a phylogeny. In this paper, we improved PMAG to introduce a better algorithm that can assemble gene orders with fewer contigs, hence provided better accuracy. This new algorithm is also faster, enabling us to handle larger datasets. Through simulation experiments, we verify the usefulness of our biased transition model and re-rooting procedure we incorporate in the program. Then the performance of PMAG is evaluated against other existing methods including InferCARsPro, GapAdj, SCJ and GASTS under a wide range of settings.

### Genome rearrangement

Given a set of 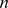 genes 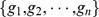, a genome can be represented by an *ordering* of these genes. To indicate the strandedness of genes, each gene is assigned with an orientation that is either positive, written 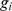, or negative, written 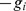. Two genes 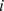 and 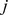 are said to be *adjacent* in genome 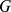 if 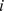 is immediately followed by 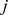, or, equivalently, 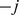 is immediately followed by 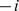. A *breakpoint* of two genomes is defined as an adjacency appears in one but not in the other.

Let 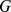 be the multi-chromosomal genome with signed ordering 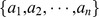, 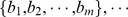 (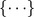
indicates a chromosome). An *inversion* (also called *reversal*) between indices 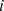 and 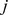 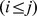 of chromosome 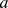, produces a chromosome 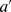 with linear ordering 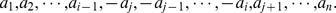



For example, the identity genome 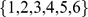 is transformed into 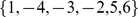 when the gene block 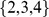 is inverted.

A *transposition* on a chromosome 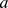 acts on three indices 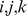, with 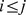 and 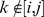, picking up the interval 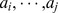 and inserting it immediately after 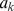. Thus the chromosome 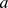 of the genome is replaced by (assume 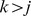): 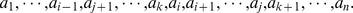



For example, genome 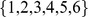 is transformed into 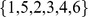 when the gene block 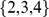 is moved in front of gene 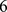.

A *translocation* on a genome 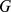 acts on two chromosomes 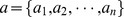 and 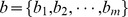. Given two indices 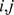, it picks up the interval 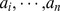 and 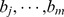 and then changes their places. Thus the two chromosomes 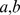 of genome 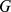 become 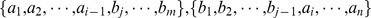



Yancopoulos [11] proposed a universal double-cut-and-join operation that accounts for inversions, transposition and translocations which resulted in a new genomic distance that can be computed in linear time. In particular, a DCJ operation consists of cutting two connections (breakpoints) in the genome, and rejoining the resulting four unconnected ends in two new pairs. Although there is no direct biological evidence for DCJ operations, these operations are very attractive because they provide a simpler and unifying model for genome rearrangement.

Later the Single-Cut-or-Join (SCJ) [12] operation was proposed as a basis for a new rearrangement distance between multi-chromosomal genomes, leading to very fast algorithms. The SCJ operation is modeled on the two most fundamental rearrangement operations—the cutting and joining of adjacencies. A cutting operation breaks an adjacency into two telomeres, and a joining operation is performed in the opposite way by pairing two telomeres into an adjacency. Any cutting or joining applied to the genome will be called a Single-Cut-or-Join (SCJ) operation. Since the genome is represented as a set of adjacencies, a cutting can also be viewed as the removal of an adjacency from the set while the joining is the addition of an adjacency.

Event-based methods typically iterate over each internal node to solve for the median genomes until the sum of rearrangement events over all edge distances (tree score) is minimized. The median problem can be formalized as follows: given a set of 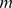 genomes with permutations 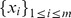 and a distance measurement 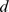, find another permutation 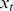 such that the median score defined as 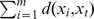 is minimized. However solving even the simplest case when 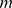 equals to three is NP-hard for most distance measurements [13], [14]. Among all existing median solvers, the best is the DCJ median solver proposed by Xu and Sankoff (ASMedian [15]) based on the concept of adequate sub-graph and decomposes a multiple breakpoint graph [16] into smaller and easier graphs. Although ASMedian could remarkably scale down the computational expenses of median searching, it yet runs very slow when the genomes are distant. Based on ASMedian, Xu developed its heuristic algorithm GASTS that can quickly score a fixed phylogenetic tree and enables us to attack previously unapproachable problems by GRAPPA and MGR, as demonstrated on a set of vertebrate genome with over 2,000 genes.

### Ancestral gene-order reconstruction based on gene adjacencies

Over recent years, a collection of models based on the study of gene adjacencies have been proposed for solving various rearrangement problems. In these models, each gene adjacency is considered as a binary character and the problem of reconstructing ancestral genomes can then be reduced as deciding, for every adjacency, whether an ancestral genome contains the adjacency.

The breakpoint-like model, single-cut-or-join (SCJ), utilized the Fitch's algorithm to reconstruct ancestors based on binary characters in terms of gene adjacencies; however, the characters are not independent, since conflicting adjacencies cannot belong simultaneously to the same genome. The SCJ's strategy of solving the conflict is simply to initialize the root with absence thus any ambiguity state will be resolved at the root as absence. This method is regarded as the only known distance for which the ancestral genome reconstruction problem has a polynomial time solution [8].

The other type of model that handles gene adjacencies relies on two separate steps. First, the weight or probability that a gene adjacency is present in a genome is computed independently. Then those gene adjacencies are assembled into a valid ancestral genome. InferCAR [17] and its probabilistic version InferCARsPro are the pioneering methods based on this model. In this model, all combinations of gene adjacencies are considered, and their probabilities are computed by a variant of the Fitch's parsimony algorithm. Finally, a greedy heuristic is used for to assemble the genes into a valid genome.

Later by relaxing the constraint of gene adjacency to gapped adjacency, GapAdj is proposed with the computation of a rigorous score for each potential ancestral adjacency 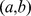, reflecting the maximum number of times 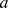 and 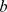 can be adjacent for any setting of ancestral genomes, as well as an algorithm to generate more reliable amount of chromosomes. Simulation experiments conducted by GapAdj show that GapAdj often ended up with a completely assembled genome, but resulted in a higher error rate that InferCAR.

## Algorithmic details

Our recent method *Probabilistic Method of Ancestral Genomics* (PMAG) [1] is also based on adjacencies and uses a probabilistic framework. It requires a given topology of the input genomes (assumed to be the phylogeny) and places the known genomes at the leaves. PMAG first encodes the gene orders into binary sequences and estimates the parameters in the transition model for adjacency changes. It then checks each ancestral (internal) node in turn, and each will be computed independently by going through the following three steps: it first re-roots the input tree to have the target ancestor as the root of a new tree; it then uses a probabilistic inference tool to compute the conditional probabilities of all adjacencies; at last it uses a greedy algorithm similar to that presented in [17] to assemble a valid gene order. The last step is very critical, but the greedy algorithm tends to produce excessive number of contigs, indicating that it is very easy to get trapped in local optima. In this paper, we introduce a new assembly algorithm, which not only improves the accuracy of the assembled gene orders, but also reduce the number of contigs to be very close to the true result. The following are the details of our algorithm.

### Re-rooting the phylogeny

**Figure 1:**
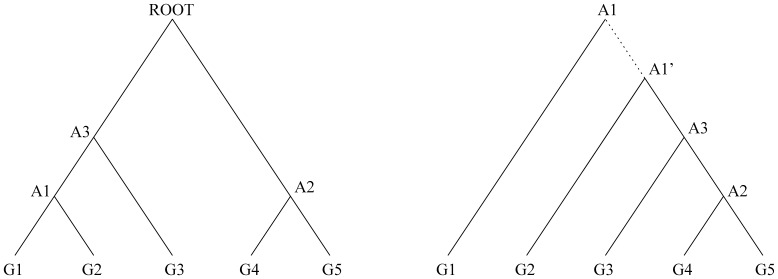
Re-rooting the phylogeny tree from the original root to the ancestral node under inference which is 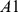 in this case. Auxiliary node 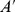 is added to preserve its binary structure.

Before we can infer the ancestral genome of an internal node, we must first re-root the given phylogeny tree to that node, making it the root of the new tree, which is a standard procedure and has already been used in [1], [9]. The underlying rationale is that the calculation of probabilities follows a bottom-up manner such that only the species in the sub-tree of the target node are considered, it will result in loss of information if the node is not the root. As we are dealing with binary trees, the re-rooting procedure will need some extra work to preserve the tree structure as demonstrated in Figure 1. In this figure, as the ancestral node we have interest in is genome 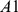, to re-root the tree on this genome, we have to add an auxiliary node 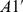, but set the branch length between 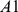 and 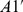 (dashed edge) as always 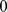.

### Obtaining probabilities of adjacencies

A gene order can be expressed as a sequence of adjacency information that specifies the presence or absence of all the adjacencies [18], [19]. Denote the head of a gene 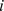 by 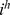 and its tail by 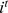. We refer 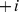 as an indication of the direction from head to tail (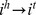) and otherwise 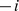 as (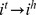). We further write 1 (0) to indicate the presence (absence) of the adjacency and we consider only those adjacencies and telomeres that appear at least once in the input genomes.

Since we are handling binary sequences with two characters, we use a general time-reversible framework to simulate the transitions from presence (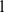) to absence (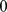) and vice versa. Since each genome contains 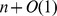 adjacencies and telomeres where 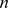 is the gene number and 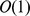 equals to the number of linear chromosomes in the genome, thus the probability that an adjacency changes from presence (1) to absence (0) in the sequence is 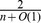 under one operation. Since there are up to 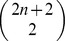 possible adjacencies and telomeres, the probability for an adjacency changing from absence (0) to presence (1) is 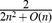. Therefore, we come to the conclusion that the transition from 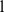 to 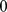 is roughly 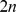 times more likely than that from 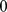 to 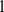.

To show how the transition model and the re-rooting procedure can respectively influence the performance of PMAG, we compare PMAG to its three variants through simulations (see details later):

- Naive: The naive version of PMAG with a neutral model of adjacency changes and fixed tree topology for all ancestral nodes.
- Naive+Model: Naive method cooperating with the biased transition model.
- Naive+Re-rooting: Naive method cooperating with the re-rooting procedure.

**Figure 2:**
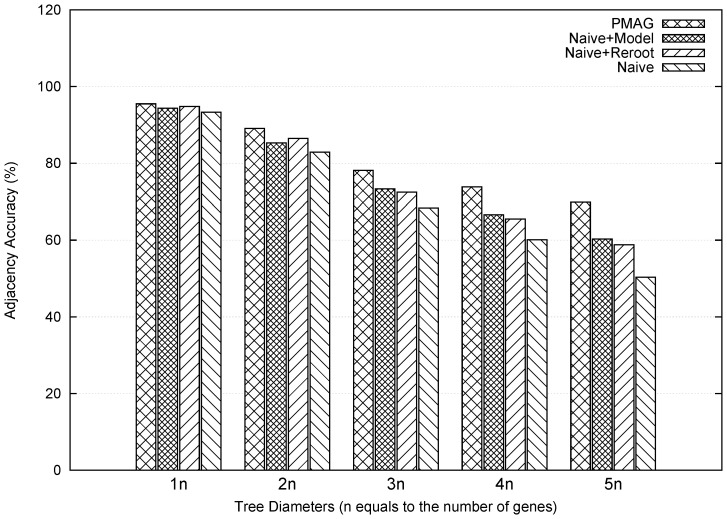
Comparison of adjacency accuracy between PMAG and its three premature versions. Datasets were simulated to have 10 genomes and 500 genes. X-axis represents the tree diameters from 1 to 5 times the number of genes.

Figure 2 summarizes the comparison result using the smaller datasets among the four methods with tree diameters from 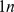 (easy case) to 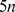 (very difficult). In general, higher tree diameter effectively increased the difficulties and hence reduced the portion of correct adjacencies all methods can recover. Unsurprisingly the Naive method is the least accurate in all cases, and both Naive+Model and Naive+Re-rooting can independently enhance the accuracy of Naive method. By incorporating both mechanisms, PMAG not only inherited both improvements, but also obtained additional improvements as well, suggesting the transition model and the re-rooting procedure be useful and indispensable for our method.

### Computing probabilities of gene adjacencies and assemble gene orders

Once we have the tree topology and binary sequences encoding the input gene orders, we use the extended probabilistic approach for sequence data described by Yang [20] to infer the ancestral gene orders at the root node, as described in detail in [1].

In the binary sequences, each site 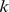 represents an adjacency with character either 0 (absence) or 1 (presence); for each site at the root node, we seek to calculate the conditional probability of observing that adjacency. Suppose 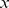 is the root of a given tree, then the conditional probability that node 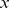 has the character 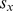 at site 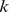, given 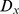 representing the observed data at site 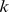 in all leaves of the sub-tree rooted at 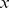, is 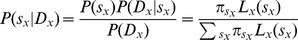
where 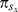 is the character frequency for 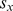.

For a site 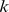, its conditional probability in the form of 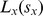 is defined as the probability of observing the leaves that belong to the sub-tree rooted at 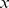, given that the character of site 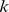 at node 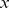 is 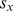. It can be calculated recursively in a post-order traversal fashion suggested by Felsenstein [1], [21] as: 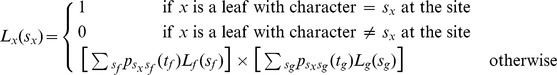
where 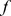 and 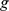 are the two direct descendants of 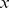. 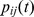 defines the transition probability that character 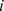 changes to 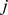 after an evolutionary distance 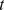. As the true branch lengths are not available, we take advantage of the widely-used maximum-likelihood estimation from the binary sequences at the leaves to estimate the branch length.

Following the deduction of transition probability in [21], our transition-probability matrix can be written as 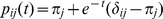



Here the 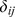 is 1 if 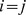, otherwise 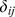 is 0.

We use RAxML [22] as it has a method to handle binary sequences efficiently and follows the steps suggested by Yang [20]. We modified RAxML so that it takes into account the biased transition model.

Our improvement over [1] is a better algorithm to assemble gene adjacencies and telomere into a valid gene order, with the requirement that each gene appears exactly once in the ancestral genome. In general, a higher probability of the presence state implies an adjacency or telomere should be more likely to be included in the ancestor; however, the decision on choosing an adjacency or telomere cannot be solely made upon its own probability as each gene can only be selected once. In the original PMAG, ancestral adjacencies are assembled by the greedy heuristic based on the adjacency graph proposed by *Ma et al.* There are two issues with the greedy approach: 1) it can only achieve a good approximation for closely related genomes; 2) it tends to create new contigs instead of connecting genes, resulted in an excessive number of contigs. In this paper, we develop the following algorithm based on the observation from [23], i.e. it can transform the problem of obtaining gene orders from (conflict) adjacencies into an instance of Traveling Salesman Problem (TSP). Although the TSP is NP-hard, it is a widely studied problem with very good solvers exist.

Specifically, we will transform genes into cities and adjacency probabilities into edge weights, and our goal is to find a tour that traverses all genes with the largest combined probabilities along the tour. As most TSP solver aims at finding a tour with minimum cost, to use probabilities as edge weights, we convert them by taking their logarithmic values. Suppose for an ancestral node 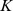 and a set of 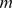 adjacencies 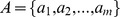 and 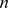 telomeres 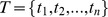 from leaf species, each with probabilities 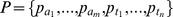, we can create the TSP graph 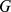 by first splitting each gene 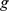 to two cities, denoted as 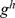 and 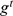 respectively, and representing each telomere 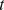 by a unique vertex 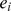, where 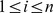. To ensure a valid tour, we must connect 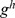 and 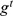 in a tour; thus we set the cost between 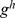 and 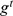 as 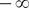. For any adjacency 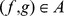, we add an edge between 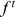 and 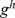; similar edges are added for other combinations of orientations 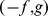, 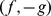 and 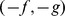, as well as genes connecting to telomeres. For the rest of edges, as we could not find a valid probability, it means these edges should have a very low chance to be present in the ancestral genome; thus we set the edge weights to 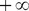 to exclude them from the solution. In the solution path, multiple contiguous caps are shrunk into a single one, and a gene segment between two caps is taken as a chromosome.

One of the best and most used TSP solver is Concorde [24], which we integrated it into MAG. For the solution path, multiple contiguous extremities are shrunk to a single one and a gene segment between two extremities is taken as a contig. Our construction of TSP topology is in a spirit similar to GapAdj, however GapAdj requires additional procedures and parameters to adjust the contig number. Instead, our inference of the ancestral genome is uniform and directly from the solution of TSP, minimizing the risk of introducing artifacts.

## Experimental Results

### Experimental design

Since actual ancestors are rarely known for sure, it is difficult to evaluate ancestral reconstruction methods with real datasets. In order to carry out a complete evaluation over a group of methods under a wide range of configurations, we conducted a collection of simulation experiments following the standard steps of such tests that have been extensively adopted in genome rearrangement studies [19], [25].

In particular, a group of tree topologies were first generated with respect to the expected tree diameters. An initial gene order was assigned at the root so it can evolve down to the leaves following the tree topology mimicking the natural process of evolution, by carrying out a number of predefined evolutionary events. In this way, we obtained the complete evolutionary history of the model tree and the whole set of genomes it has.

Normally we utilized the simulator proposed by Lin *et al.* [26] to produce birth-death tree topologies. Since SCJ has its own simulator, we used that simulator for a fair comparison in the tests involving SCJ. With a model tree, we can produce genomes of any size by simply adjusting four main parameters: the number of genomes 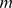, the number of chromosome 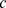, the number of genes 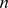, and the tree diameter 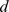 (equivalent to the branch length 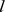 in SCJ's simulator). For the following simulation experiments, we generated datasets of two different sizes. First we generated a smaller datasets with 10 genomes, each with 500 genes and five chromosomes to closely mimic the rearrangement scenarios in bacterial genomes with multi-chromosomes. We also produced datasets of larger size contains 20 genomes, each with 2,000 genes and five chromosomes. Along each branch, we performed 80% random inversions and 20% random translocations to account for intra- and inter-chromosomal rearrangements.

The following three measurements were used to assess the predicted ancestral genomes. We first calculated the adjacency accuracy 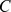 as the total number of correctly inferred adjacencies (i.e. those also appear in the true ancestral genomes) divided by the total number of adjacencies in both true genome and predicted genome. Second, we calculated distance accuracy 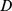 defined as the DCJ distance between a predicted ancestor and its corresponding true genome. Apparently for the genome rearrangement study, distance accuracy is more appropriate as it not only considers the adjacency changes, but also takes differences in genome structures into account. Finally, to assess the assembly capabilities, we computed assembly accuracy 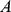 as the absolute differences of the number of chromosomes between a predicted ancestor and its corresponding truth. For each dataset, the average of each measurement across all ancestors was computed and for each tree diameter, we produced 10 datasets and reported their average, as well as their standard deviation.

### Evaluation of PMAG against SCJ

Simulator embedded in the SCJ was used, and the measurement of difficulty became the branch length 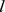, denoting the expected number of evolutionary events along an edge of the tree which is sampled from an uniform distribution on the set 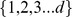, where 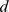 equals to 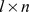 and 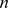 is the number of genes. As before, those events consisted of 80% of inversions and 20% translocations. Since SCJ and PMAG are both fast enough, we therefore generated a set of larger dataset containing 32 genomes, each with five chromosomes and a total of 2,000 genes.

**Figure 3:**
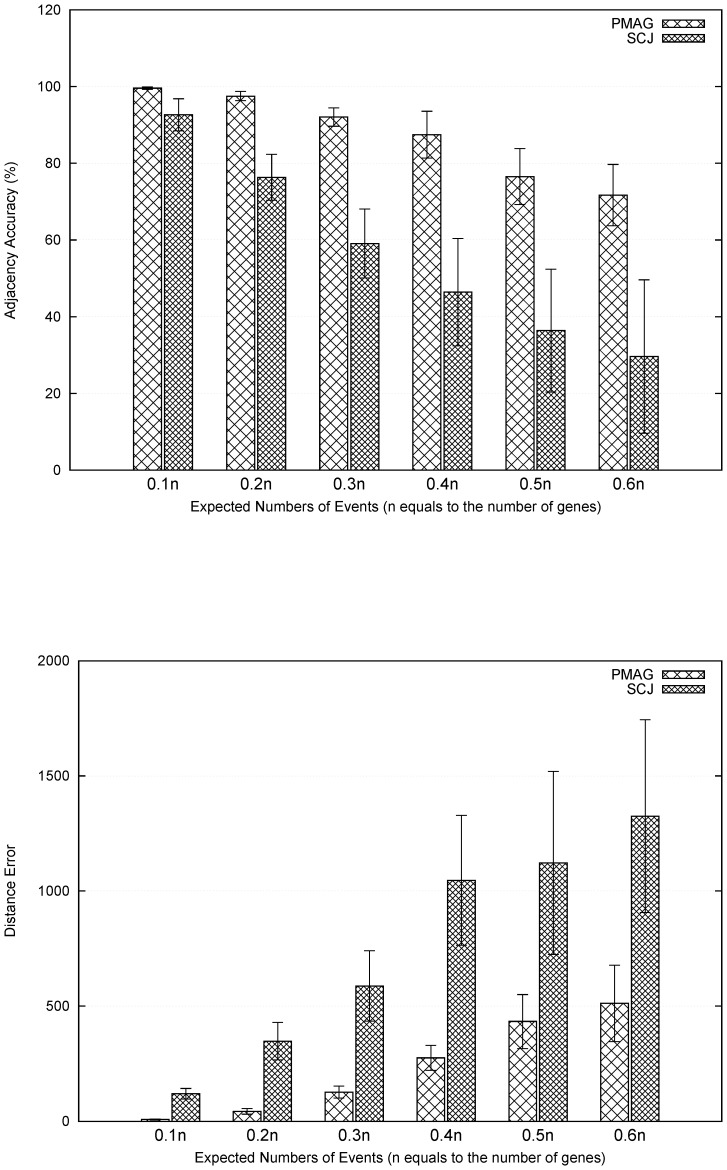
Comparison of adjacency accuracy (top) and distance accuracy (bottom) between PMAG and SCJ. Datasets were produced by the simulator provided in SCJ program that contain 32 genomes, each with five chromosomes and a total of 2,000 genes. X-axis represents the expected number of events from 0.1 to 0.6 times the number of genes.

Figure 3 demonstrates the adjacency accuracy and the distance accuracy of PMAG and SCJ respectively. This figure clearly suggests that PMAG can significantly outperform SCJ in all tested cases.

### Evaluation of PMAG against other methods

In this section, we picked three main competitors from both event-based and adjacency-based methods, and compared them with PMAG. In particular we supplied InferCARsPro with multi-chromosomal genomic distances as its branch lengths computed by GRIMM [27]. Moreover, in GapAdj, the cutoff value and maximal iterations were set to 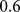 and 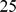 as suggested by the authors. The event-based method GASTS was simply run by providing the true tree and the input genomes. Results of InferCARsPro under large tree diameters were missing as it failed to finish the tests in three days.

**Figure 4:**
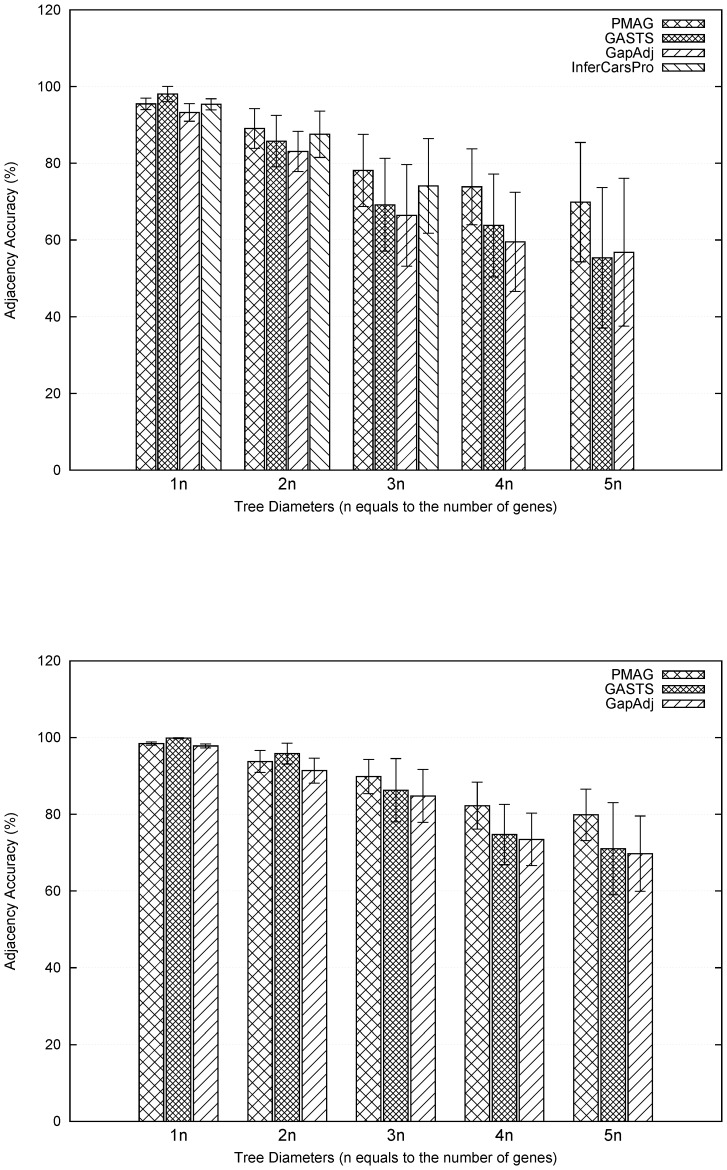
Comparison of adjacency accuracy between PMAG, InferCARsPro, GASTS and GapAdj: (top) datasets contain 10 genomes and 500 genes; (bottom) datasets contain 20 genomes and 2000 genes. Standard deviations are given at the top of bars. X-axis represents the tree diameters from 1 to 5 times the number of genes.

**Figure 5:**
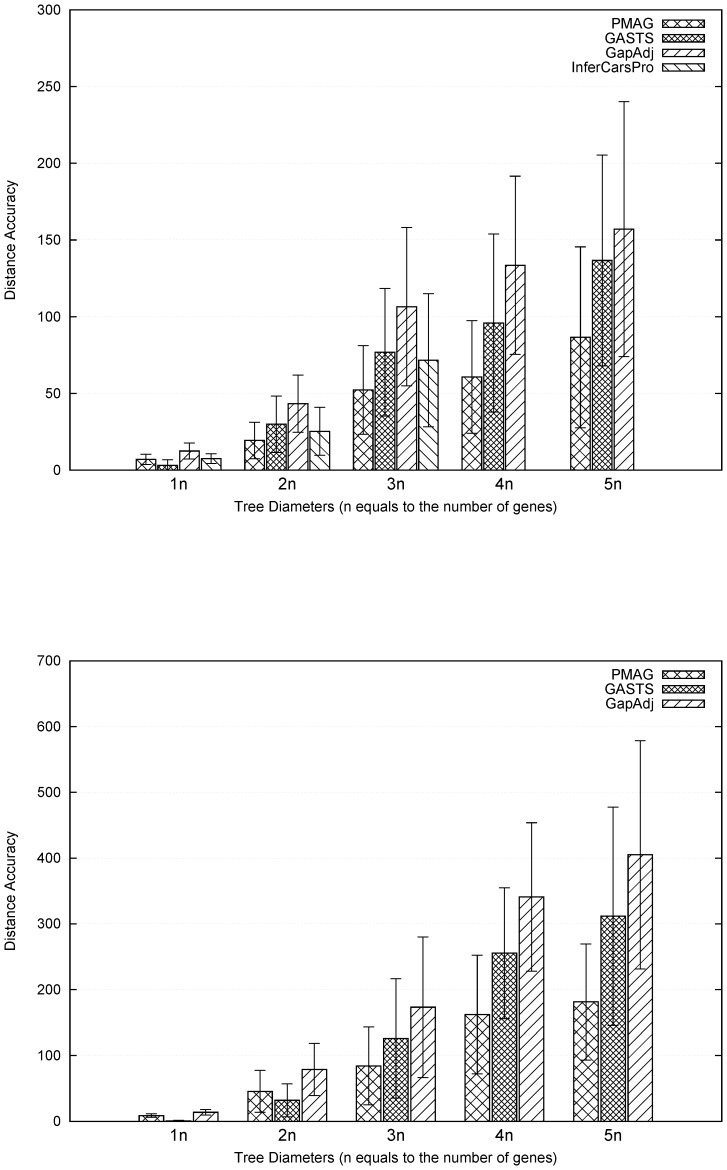
Comparison of distance accuracy between PMAG, InferCARsPro, GASTS and GapAdj: (top) datasets contain 10 genomes and 500 genes; (bottom) datasets contain 20 genomes and 2000 genes. Standard deviations are given at the top of bars. X-axis represents the tree diameters from 1 to 5 times the number of genes.

Figure 4 shows the results measured by the adjacency accuracies. When the tree diameters were 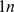, all methods were able to produce highly accurate ancestral genomes (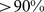) and the differences among methods were not significant. In particular, GASTS was the most accurate method, while the performances of PMAG and InferCARsPro were similar, and both were better than GapAdj. As the tree diameters went larger, GASTS quickly became unreliable which is consistent with the experimental findings reported in the study of GASTS [7]. In all tests, PMAG showed great robustness against disturbance and achieved the highest adjacency accuracy when the tree diameter grows greater than 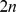. Figure 5 shows the results measured by the distance accuracies. In general, the relative performances of various methods in distance measurement are very similar to the adjacency accuracies.

### Comparison of performances on assembly

The final step of adjacency-based methods often involves assembly of adjacencies into contiguous segments which can be viewed as chromosomes or more precisely contigs. Previous methods InferCARsPro employing a greedy algorithm for assembly often ends up with an excessive number of contigs. Later the assembly accuracy was improved by GapAdj using the concept of gapped adjacencies with a sacrifice of accuracy.

**Figure 6:**
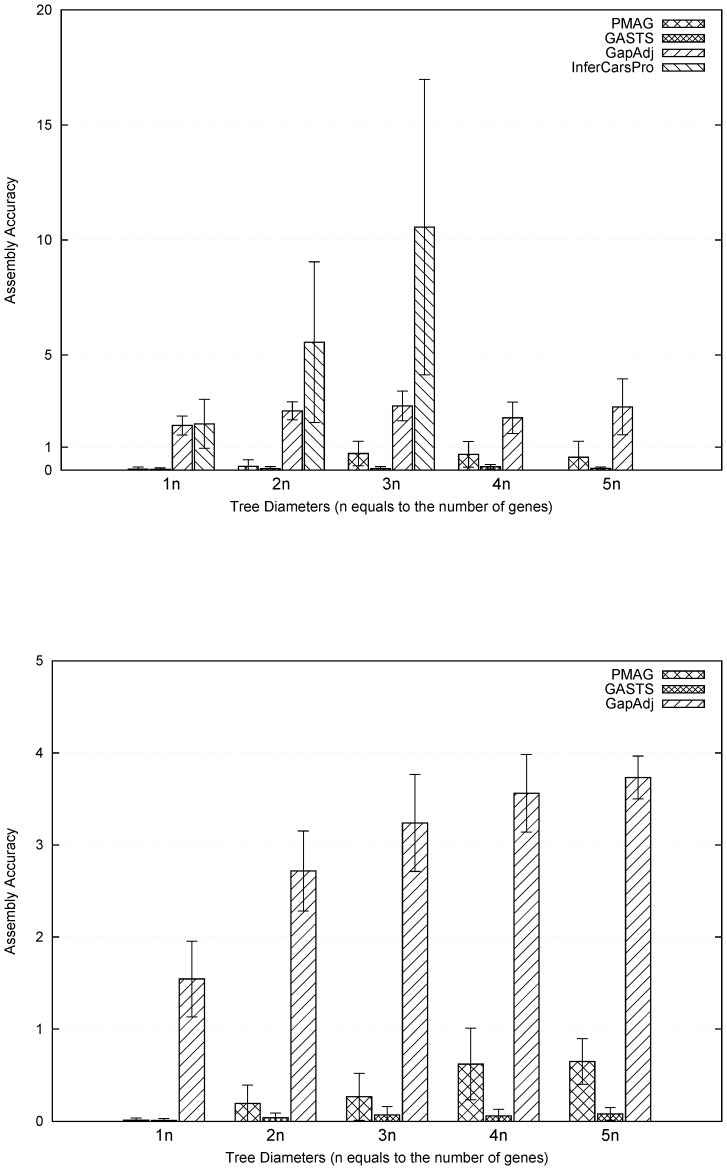
Comparison of assembly accuracy between PMAG, InferCARsPro, GASTS and GapAdj: (top) datasets contain 10 genomes and 500 genes; (bottom) datasets contain 20 genomes and 2000 genes. X-axis represents the tree diameters from 1 to 5 times the number of genes.

Our measurement of accuracy only counts adjacencies correctly recovered. However, for two assembled gene orders with similar adjacency accuracy, the one with the number of contigs close to the number of chromosomes should be viewed as having better accuracy. Thus, we summarized the number of contigs produced by various methods and computed the averages of assembly accuracy for all cases in Figure 6. From the figure, the event-based method GASTS without the need for assembly of gene adjacencies produced the most relevant number of contigs. Among the adjacency-based methods, PMAG showed much better assembly performance, and its performance was very close to GASTS. As expected, the greedy assembly used in InferCARsPro produced the least relevant number of contigs. By examining Figure 4, we found that although PMAG returned more contigs than GASTS, its distance and adjacency accuracies were better, indicating that GASTS had a tendency to introduce bad adjacencies in order to keep the number of contigs small.

Table 1 shows the assembly accuracies of PMAG and SCJ. From the table, PMAG yielded very accurate amount of contigs; however, since SCJ is overly conservative, it missed a large portion of true adjacencies and produced a massive amount of contigs. In other words, SCJ has difficulty in assembling gene orders due to its overly simplified cost to weigh adjacencies.

**Table 1:** Comparison of assembly accuracy between PMAG and SCJ under different expected numbers of evolutionary events along a tree branch. (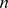 equals to the number of genes).

Tree Diameter	0.1n	0.2n	0.3n	0.4n	0.5n	0.6n
PMAG	0	0.13	0.30	0.45	0.48	0.63
SCJ	157	476	785	1031	1280	1413

### Time efficiency

All tests were conducted on a workstation with 2.4 GHz CPUs and 4 GB RAM. In general, SCJ is undoubtedly the fastest and can return results in just a few seconds. In the experiments with small datasets, InferCARsPro required the most amount of time and the other three methods can always finish within a minute.

We summarized the time consumptions of PMAG, GapAdj, GASTS and the previous greedy version of PMAG in handling large datasets in table 2. From the table, the running time of PMAG and GapAdj were very close and stable, and tree diameters did not remarkably slow down these programs. On the other hand, GASTS severely suffered from large tree diameters, suggesting its potential limitation in handling genomes that are distant to each other.

**Table 2:** Time consumptions of four methods (including the previous greedy version of PMAG in analyzing large datasets under different tree diameters. (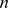 equals to the number of genes).

Tree Diameter	PMAG	GapAdj	GASTS	PMAG-Greedy
1n	13 min	10 min	1 min	1 min
2n	13 min	12 min	10 min	3 min
3n	15 min	12 min	45 min	3 min
4n	18 min	14 min	120 min	4 min
5n	20 min	16 min	159 min	5 min

### Comparing PMAG with Its Previous Version

We compared PMAG with its greedy version to evaluate the new TSP approach. Table 3 and Table 4 showed the adjacency and assembly accuracy, respectively. These tables suggested that although the TSP solver was about 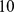 times slower than the greedy solver (Table 2), the new PMAG method had achieved improved adjacency accuracy with much better performance in term of the number of recovered contigs.

**Table 3:** Comparison of the adjacency accuracy between PMAG and its greedy version. The number of genomes is 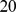 and the number of genes is 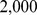.

Tree Diameter	1n	2n	3n	4n	5n
PMAG	98.7	93.5	89.7	82.2	79.5
PMAG-Greedy	98.5	93.2	88.6	80.2	77.8

**Table 4:** Comparison of the assembly accuracy between PMAG and its greedy version. The number of genomes is 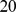 and the number of genes is 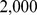.

Diameter	1n	2n	3n	4n	5n
PMAG	0.03	0.2	0.3	0.6	0.6
PMAG-Greedy	2.1	5.5	8.5	15.9	17.6

## Conclusions and Future Work

In this paper, we introduced the adjacency-based method PMAG in the probabilistic framework for ancestral gene-order inference. PMAG determines the state for each adjacency in the binary encoding to be either present or absent in an ancestral genome according to its conditional probability. Ancestral gene orders are finally assemblies by connecting individual adjacencies into continuous regions using a TSP approach Experimental results reveal that PMAG can not only accurately infer ancestral genomes, and also did a good job in assembling adjacencies into valid gene orders. Finally, PMAG is fast and also stable across a wide range of configurations.

However, much work remains to be done. As PMAG relies on gene adjacencies, how to recover adjacencies lost in evolution (thus not shown in leave genomes) is an interesting problem. In the current implementation, these adjacencies have no definite edge weight and they are all set as 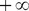. As a result, the TSP tour is prevented from passing through them, although they may be better choices. Our experiments showed that these adjacencies account for about 25% of errors in PMAG; thus, we need to devise a method that can assign better edge weights to missing adjacencies. Since each internal node can be computed independently, the speed of PMAG can be further improved by utilizing the presence of multiple computing cores in modern CPUs by placing each node's computation on a core.
